# Mass media influences on family planning knowledge, attitudes and method choice among sexually active men in sub-Saharan Africa

**DOI:** 10.1371/journal.pone.0261068

**Published:** 2022-01-27

**Authors:** Massy Mutumba

**Affiliations:** Department of Health Behavior & Biological Sciences, University of Michigan School of Nursing, Ann Arbor, Michigan, United States of America; FHI360, UNITED STATES

## Abstract

Men are underrepresented in family planning (FP) research, and despite the widespread promotion of FP through mass media, there is no systematic evaluation on how mass media exposure influences their FP knowledge, attitudes and behavior. Using Demographic and Health Survey (DHS) data from 31 countries in Sub-Saharan Africa (SSA), collected between 2010 and 2019, this paper examines the associations between three types of traditional mass media (radio, television and print) with FP knowledge, attitudes and method choices among reproductive age men in SSA, relative to other socio-cultural factors. Estimates to quantify the relative contribution of each type of mass media, relative to other evidence-based socio-cultural influences on FP outcomes, were derived using the Shorrocks-Shapley decomposition. Radio exposure had the largest impact on FP knowledge, attitudes and method choice, accounting for 26.1% of the variance in FP knowledge, followed by Television (21.4%) and education attainment (20.7%). Mass media exposure had relatively minimal impact on FP method choice, and between the three types of mass media, television (8%) had the largest influence on FP method choice. Print media had comparatively lesser impact on FP knowledge (8%), attitudes (6.2%) and method choice (3.2%). Findings suggest that mass media exposure has positive influences on FP knowledge, attitudes and method choice but its influence on FP knowledge, attitudes and method choice is smaller relative to other socio-cultural factors such as education, household wealth and marital status. As such, efforts to increase FP uptake in Sub-Saharan Africa should take into consideration the impact of these socio-cultural economic factors.

## Introduction

Family planning (FP) has numerous crosscutting benefits to individuals and society. It enables individuals or couples to fulfill their reproductive health and fertility needs, reduces maternal and infant mortality, and promotes women’s empowerment and economic development through reduced household poverty and increased participation of women in the labor force [1–3]. Within the past two decades, use of modern FP methods increased rapidly in Sub-Saharan Africa (SSA), from 17.4% in 1990 to 28.5% in 2017 [4]. The FP 2020 initiative, a global partnership to enable an additional 120 million FP users in the world’s poorest countries by the year 2020 [5, 6], re-invigorated efforts towards realizing universal access to effective FP methods, which would avert over 36 million abortions, 70,000 maternal deaths, and 52 million unintended pregnancies [1]. Despite these efforts, unmet need for FP–defined as women who wish to delay or avoid childbearing who are still not using any FP method–remains high: it is estimated that up to 25% of women in SSA have an unmet need for FP [4].

Numerous studies have examined the determinants of FP use in SSA [7–11]. Higher education attainment and household wealth and lower fertility aspirations increase FP uptake [8, 10, 11]. The most frequently reported barriers to modern FP use: lack of knowledge or access to FP, fear of side effects and health concerns related to modern FP methods, lack of decision-making autonomy and partner opposition to FP, including fears of infidelity [9]. Despite the recognition that men’s involvement is essential to the success of FP programs [12–15], men’s voices and preferences are largely unheard in FP research. With a few notable exceptions, the expansive literature on FP in SSA largely focuses on women’s experiences and their proxy reports on their partners’ FP preferences, attitudes and behavior. While this relationship is evolving, men still play an important role in influencing women’s fertility and FP intentions and behaviors [16–20]. Studies have shown that when men are educated about reproductive health issues, they are more likely to be involved in reproductive decision-making. However, most interventions on men’s involvement target HIV and intimate partner violence. While the interventions offer relevant cross-cutting lessons, the limited research on men’s FP behavior–including their knowledge, attitudes, method preferences and choices limits curtails efforts to increase men’s uptake of modern FP methods.

Despite the expanded access to the internet, most countries in SSA still rely on traditional mass media such as radio, television and print to mobilize and sensitize communities on a range of health issues including FP. Mass media can affect FP attitudes and behavior by providing new information and alternative forms of behavior, or alter ideation pathways by shaping a consumer’s aspiration and self-identity [21–25]. Indeed, evidence from previous studies indicates that mass media exposure can increase knowledge and uptake of FP, even among men [23, 25–32]. However, the impact of mass media exposure on FP knowledge, attitudes and behavior is not always consistent. For example, a study conducted among men aged 15–59 years in select cities of three African countries (Kenya, Nigeria and Senegal) found that mass media exposure to FP demand-generating program activities increased modern FP use among men in Kenya and Senegal, but not Nigeria [26]. Another study conducted in Bolivia found that mass media campaigns increased FP knowledge and use but did not have any impact on FP attitudes [30]. Given the sparse literature on FP among men and the inconsistency of findings on the effect of mass media exposure on FP use, this paper draws on nationally representative data from 31 countries in SSA to provide an expansive view on the effects of mass media exposure on FP. More specifically, this paper draws on nationally representative data to estimate the impact of the different types of traditional mass media (radio, television and print) on FP knowledge, attitudes and method choices among reproductive age men in SSA, relative to socio-demographic factors such as education and household wealth.

## Methods

### Data sources

Data are drawn from Demographic and Health Surveys (DHS) collected in 31 countries in SSA, between 2010 and 2019. DHS are nationally representative household surveys that collect information on varied topics including FP in low- and middle-income countries. The surveys are implemented by the local government entities or universities, with technical assistance from ICF through the DHS program [33]. Data are collected using standardized procedures, methodologies and manuals, including standardized model questionnaires that countries are asked to adopt in their entirety [34]. Briefly, DHS uses a two-stage random sampling approach: 1) selection of enumeration areas (EAs) using probability proportional to size and power allocation with adjustments to meet the minimum number of clusters per survey domain required for a DHS survey; and 2) selection of households within EAs. All men aged 15 and older who were either permanent residents of the selected households or visitors who stayed in the household the night before the survey were eligible to be interviewed using a standardized man’s questionnaire. This uniformity in procedures ensured comparability of data across countries, thus enabling a pooled data approach to examining the influence of mass media on FP knowledge, attitudes and behaviors. The 31 countries included in these analyses are listed in **Table 1**, along with their sample characteristics. Our analyses focus on sexually active men because they may be motivated to use FP methods to prevent pregnancy.

**Table 1:** Summary of survey characteristics for countries included in the analyses.

Country	Survey year	Sample size	Percent
Angola	2015–2016	5,684	3.28
Burkina Faso	2010	7,307	3.67
Benin	2017–2018	7,595	3.97
Burundi	2016–2017	7,552	3.31
Congo-Democratic Republic	2013–2014	8,656	4.79
Cote d’Ivoire	2011–2012	5,135	2.82
Cameroon	2018–2019	6,978	3.56
Comoros	2012	2,167	1.09
Ethiopia	2,015	12,688	5.75
Ghana	2014	4,388	2.22
Gambia	2013	3,821	1.65
Guinea	2018	4,117	2.06
Kenya	2014	12,819	6.8
Liberia	2013	4,118	2.27
Lesotho	2014	2,931	1.61
Malawi	2015	7,478	4.06
Mali	2018	4,618	2.32
Mozambique	2011	4,035	2.35
Namibia	2013	4,481	2.43
Nigeria	2018	13,311	6.21
Niger	2012	3,928	1.82
Rwanda	2014–2015	6,217	2.93
Senegal	2017	6,977	2.87
Sierra Leone	2013	7,262	4
South Africa	2016	3,618	2.02
Chad	2014–2015	5,248	2.53
Tanzania	2015–2016	3,514	1.8
Togo	2013–2014	4,476	2.29
Uganda	2016	5,336	2.83
Zambia	2018	12,132	6.55
Zimbabwe	2,015	8,396	4.16
Total		207,782	100.00

### Protection of human subjects

This paper utilizes publicly available and de-identified data, which do not require ethical approval. Government agencies or universities collect DHS data, and are responsible for securing ethical clearance from the responsible entities in each country and ensuring compliance with procedures to protect study participants. As per DHS study protocols, each participant is required to provide informed consent prior to participating in the survey.

### Variables

#### Outcomes

Three outcome variables are explored: FP knowledge, attitudes and method choice. FP knowledge, a binary indicator, was defined as knowledge of any modern FP method (mv301). FP attitudes were derived from two items: mv3b25a: ‘FP is a woman’s business, man should not worry’, and mv3b25b: ‘women who use FP become promiscuous’. Responses are measured on a 3-item scale–disagree, agree and don’t know. For analytic purposes, these items are analyzed separately and responses were recoded into a binary yes/no indicator. Lastly, FP method choice was derived from variable mv313 –current FP by method type. Responses were recoded into three categories: none/traditional, women’s (partner) method (e.g. pill, injectables, IUD) and men’s methods (i.e. men’s condom or sterilization).

#### Predictor variables

The primary predictor variable was mass media exposure defined as frequency of exposure to radio, television, and print (i.e., newspapers or magazines). Frequency of mass media exposure was derived from responses to the questions: (1) Do you read a newspaper almost every day, at least once a week, less than once a week, or not at all? (2) Do you listen to the radio almost every day, at least once a week, less than once a week, or not at all? and (3) Do you watch television almost every day, at least once a week, less than once a week, or not at all? For each type of mass media, responses were recoded into three categorical levels, ranging between 0–2: low exposure (i.e. not at all), medium exposure (i.e. less than once a week) and high exposure (at least once a week/almost every day).

#### Covariates

Socio-demographic indicators were based as covariates in the analyses and these included: (1) birth cohort measured in 10 year increments; (2) education attainment defined as none, primary, secondary and tertiary or higher; (3) place of residence i.e. urban or rural; (4) marital status defined as never married, currently married/cohabiting and previously married; (5) total number of children ever born–categorized as none, 1–4 children and 5 or more; (6) employment status defined as binary variable of whether the responded is currently working or not; and (7) household wealth quintiles computed from a composite measure of a household’s cumulative living standard, determined via analysis of data on ownership of selected household assets such as televisions, bicycles, materials used for housing construction, and types of water access and sanitation facilities [35]. Country and survey year were also included as covariates to account for unmeasured country features that may influence mass media and FP behavior (e.g. mass media regulation and intensity of family planning program) and variations in the timing of data collection.

#### Analyses

Analyses were conducted in STATA (v.16), using the complex samples analysis ‘*svy*’ command to account for the complex sampling design. The sample was limited to sexually active men. First, univariate and bivariate analyses were conducted to characterize the distribution of the outcomes, predictor variables and covariates (**Table 2**), and to examine the associations between FP knowledge, attitudes and method choice with each type of mass medium and covariate. Subsequently, multivariable regressions–logistic regressions for FP method and attitudes and multinomial logistic (mlogit) regressions for FP method choice–were used to assess the influence of each type of mass media on FP knowledge, attitudes and behaviors (**Table 3**). To quantify the relative contribution of each type of mass media, the STATA module–shapley2, a post-estimate module–was used to compute the Shorrocks-Shapley decomposition of any statistic of the model, typically the R squared (R^2^) or pseudo R^2^ for logistic regressions. Because the shapley2 module cannot work with mlogit regressions, a series of independent logistic regressions were conducted to estimate the relative contribution of mass media on using no method or traditional method, woman (partner) method and man method (**Table 4**). In supplemental analyses, these analyses were repeated at the country level, to assess their generalization to specific countries. Our results highlight the relative contributions of the mass media exposure along with other competing factors that can be leveraged to increase men’s engagement and uptake of FP.

**Table 2:** Descriptive summary of study variables.

*Outcomes*	*Percent*	*Covariates*	*Percent*
*FP knowledge*		Birth cohort	
Knows at least one modern FP method		1945–1955	1.6
No	2.7	1956–1965	11.0
Yes	97.3	1966–1975	20.9
*FP attitudes*		1976–1985	29.2
FP is a woman’s business		1986–1995	29.4
Disagree	73.6	1996–2005	7.9
Agree/don’t know	26.4	Education attainment	
A woman who uses FP becomes promiscuous		None	21.7
Disagree	61.2	Primary	30.4
Agree/don’t know	38.8	secondary	38.0
*FP behavior*		Tertiary	9.9
FP method choice		Place of residence	
None or traditional	64.9	Urban	41.9
Women’s (partner) method	14.7	Rural	58.1
Men’s method	20.4	Household wealth quintiles	
***Correlates of mass media exposure***		Poorest	16.5
Frequency of listening to radio		Poorer	18.3
Not at all	22.8	Middle	19.5
Less than once a week	19.5	Richer	21.4
At least once a week	57.7	Richest	24.4
Frequency of watching TV		Marital status	
Not at all	42.3	Never married	28.1
Less than once a week	18.3	Married/cohabiting	67.3
At least once a week	39.4	Previously married	4.6
Frequency of reading a newspaper or magazine		Employment	
Not at all	63.3	Not working	14.1
Less than once a week	16.5	Currently working	85.9
At least once a week	20.2	Total children ever born	
		None	29.3
		1–4 children	41.6
		5+ children	29.1

**Table 3:** Multivariable logistic regression examining the associations between mass media exposure and FP knowledge, attitudes and method choice among sexually active men in sub-Saharan Africa.

	*Know modern FP method*	*FP is woman’s business*	*Women who use FP become promiscuous*	*Women’s (partner) methods*	*Men’s methods*
Frequency of listening to radio ^a^					
Medium	2.37***	0.81***	0.95**	0.99	1.04
	(2.09–2.68)	(0.77–0.85)	(0.90–0.99)	(0.91–1.06)	(0.98–1.11)
High	3.23***	0.75***	0.93***	1.10***	0.99
	(2.89–3.61)	(0.72–0.78)	(0.89–0.97)	(1.03–1.17)	(0.94–1.05)
Frequency of watching television ^a^					
Medium	2.63***	0.83***	0.91***	1.21***	1.34***
	(2.21–3.13)	(0.78–0.87)	(0.87–0.96)	(1.14–1.29)	(1.26–1.41)
High	3.18***	0.90***	1.01	1.23***	1.63***
	(2.65–3.83)	(0.86–0.95)	(0.96–1.06)	(1.15–1.31)	(1.54–1.73)
Frequency of reading newspapers ^a^					
Medium	1.49***	0.83***	0.82***	1.42***	1.22***
	(1.20–1.86)	(0.79–0.87)	(0.79–0.86)	(1.34–1.51)	(1.16–1.29)
High	1.11	1.06*	0.88***	1.62***	1.33***
	(0.88–1.40)	(1.00–1.13)	(0.84–0.93)	(1.52–1.72)	(1.26–1.41)
Education ^b^					
Primary	2.54***	0.79***	0.81***	2.25***	2.01***
	(2.24–2.87)	(0.76–0.83)	(0.78–0.85)	(2.08–2.43)	(1.88–2.15)
Secondary	2.36***	0.66***	0.80***	1.88***	2.56***
	(1.95–2.86)	(0.62–0.70)	(0.76–0.85)	(1.72–2.05)	(2.38–2.74)
Tertiary	7.28***	0.40***	0.58***	1.97***	2.84***
	(4.32–12.27)	(0.36–0.44)	(0.54–0.63)	(1.75–2.22)	(2.59–3.12)
Birth cohort ^c^					
1956–1965	0.61***	1.39***	1.44***	1.17	1.66***
	(0.45–0.84)	(1.21–1.60)	(1.27–1.62)	(0.93–1.47)	(1.21–2.27)
1966–1975	0.72**	1.51***	1.52***	1.78***	2.36***
	(0.53–0.99)	(1.32–1.73)	(1.35–1.71)	(1.42–2.23)	(1.73–3.21)
1976–1985	0.71**	1.55***	1.56***	1.85***	2.93***
	(0.52–0.98)	(1.35–1.78)	(1.38–1.76)	(1.48–2.32)	(2.16–3.98)
1986–1995	0.57***	1.81***	1.94***	1.60***	3.17***
	(0.41–0.80)	(1.57–2.09)	(1.71–2.20)	(1.27–2.02)	(2.32–4.32)
1996–2005	0.41***	2.53***	2.45***	0.58***	2.60***
	(0.28–0.59)	(2.17–2.96)	(2.14–2.80)	(0.43–0.76)	(1.89–3.57)
Place of residence ^d^					
Rural	0.76***	0.77***	0.83***	1.55***	0.78***
	(0.64–0.91)	(0.72–0.82)	(0.78–0.87)	(1.46–1.66)	(0.73–0.82)
Household wealth ^e^					
Poor	1.26***	0.91***	0.92***	1.25***	1.16***
	(1.12–1.43)	(0.87–0.96)	(0.88–0.96)	(1.17–1.34)	(1.09–1.24)
Middle	1.20***	0.83***	0.86***	1.37***	1.22***
	(1.05–1.37)	(0.79–0.88)	(0.82–0.91)	(1.27–1.47)	(1.15–1.31)
Richer	1.17*	0.73***	0.78***	1.67***	1.19***
	(0.99–1.39)	(0.69–0.78)	(0.74–0.83)	(1.54–1.81)	(1.11–1.28)
Richest	1.17	0.58***	0.63***	1.82***	1.15***
	(0.94–1.45)	(0.54–0.63)	(0.59–0.68)	(1.66–1.99)	(1.06–1.24)
Employment ^f^					
Currently working	1.99***	0.81***	0.92***	0.98	0.97
	(1.68–2.35)	(0.77–0.85)	(0.88–0.96)	(0.91–1.05)	(0.92–1.02)
Marital status ^g^					
Married/cohabiting	0.55***	0.90***	0.79***	1.06	0.15***
	(0.45–0.67)	(0.84–0.96)	(0.75–0.84)	(0.96–1.16)	(0.14–0.16)
Previously married	0.59***	1.08*	1.17***	0.66***	0.64***
	(0.46–0.76)	(0.99–1.19)	(1.09–1.27)	(0.57–0.76)	(0.59–0.70)
Total number of children ever born ^h^					
1–4	1.63***	0.95	0.96	2.84***	1.19***
	(1.38–1.92)	(0.90–1.01)	(0.91–1.01)	(2.57–3.14)	(1.11–1.27)
5+	1.15	1.04	1.08**	2.25***	0.94
	(0.95–1.39)	(0.97–1.12)	(1.02–1.15)	(2.01–2.52)	(0.85–1.03)
country	1.01***	1.01***	1.01***	1.04***	1.01***
	(1.01–1.02)	(1.01–1.01)	(1.00–1.01)	(1.04–1.04)	(1.01–1.01)
year	1.07***	1.01	1.01***	0.95***	0.95***
	(1.05–1.10)	(1.00–1.02)	(1.00–1.02)	(0.94–0.95)	(0.94–0.96)
Observations (N)	166,643	156,724	156,716	158,690	158,690
	0.1252	0.0277	0.0167	0.0647	0.1755

Notes: *** p<0.01,

** p<0.05,

* p<0.1

^a^ Reference group is low frequency;

^b^ Reference is no education;

^c^ Reference is birth cohort 1945–1955;

^d^ Reference is urban place of residence’

^e^ Reference is poorest;

^f^ Reference is currently unemployed;

^g^ Reference is never married;

^h^ Reference is no children.

**Table 4:** Relative contribution of exposure to mass media and other relevant covariates to FP knowledge, attitudes and method choice among sexually active men in sub-Saharan Africa.

	Know modern FP method		FP is woman’s business		Women who use FP become promiscuous		No method or traditional FP method		Women’s (partner) method		Men’s method	
Factor	Shapley value	%	Shapley value	%	Shapley value	%	Shapley value	%	Shapley value	%	Shapley value	%
Radio	0.033	26.1	0.004	15.2	0.001	5.2	0.001	2.9	0.004	5.61	0.001	0.5
Television	0.027	21.4	0.002	7.7	0.001	2.9	0.010	11.3	0.001	0.99	0.014	8.0
Newspaper	0.010	8.0	0.002	6.2	0.002	10.7	0.009	10.7	0.005	8.02	0.006	3.2
Education	0.025	20.1	0.008	29.7	0.003	19.9	0.022	24.6	0.005	7.13	0.022	12.4
Birth cohort	0.008	6.4	0.003	10.0	0.004	21.8	0.013	14.2	0.004	5.95	0.040	22.9
Residence	0.006	4.4	0.001	3.2	0.001	2.7	0.003	3.8	0.002	3.2	0.009	4.9
Wealth	0.007	5.5	0.007	23.5	0.004	23.0	0.007	7.5	0.003	4.96	0.005	2.9
Employment	0.007	5.7	0.002	7.7	0.001	4.7	0.002	2.3	0.004	6.01	0.009	4.8
Marital status	0.001	1.1	0.001	3.5	0.002	9.3	0.015	16.7	0.017	26.62	0.065	36.9
Country	0.001	1.1	0.001	2.0	0.001	3.2	0.012	13.4	0.025	39.12	0.006	0.3
Survey year	0.003	2.6	0.001	1.2	0.001	1.9	0.002	2.4	0.001	1.14	0.002	1.3
Total	0.125	100	0.028	100	0.017	100	0.089	100	0.065	100	0.176	100
Pseudo R2 (%)	12.52		2.77		1.67		8.88		6.47		17.55	

## Results

### Descriptive summary of FP and mass media exposure

**Fig 1:**
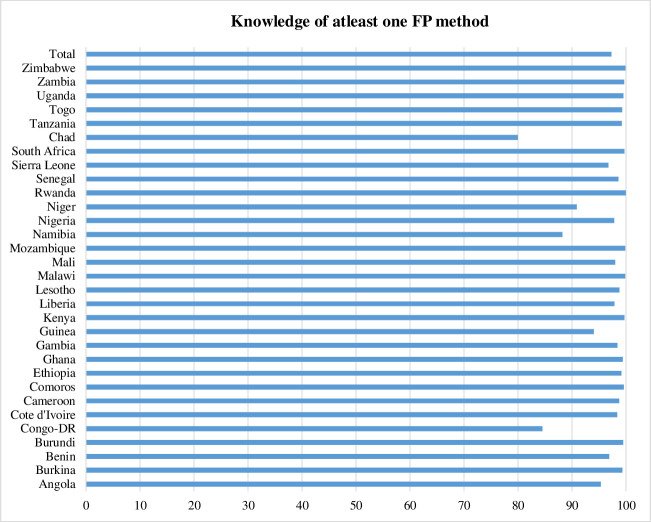
FP knowledge among reproductive age men in Sub-Saharan Africa, by country.

**Fig 2:**
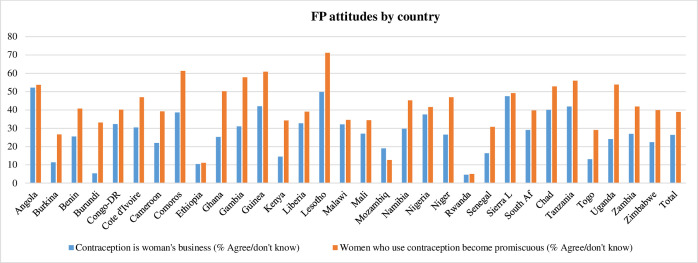
FP use attitudes among reproductive age men in sub-Saharan Africa, by country.

**Fig 3:**
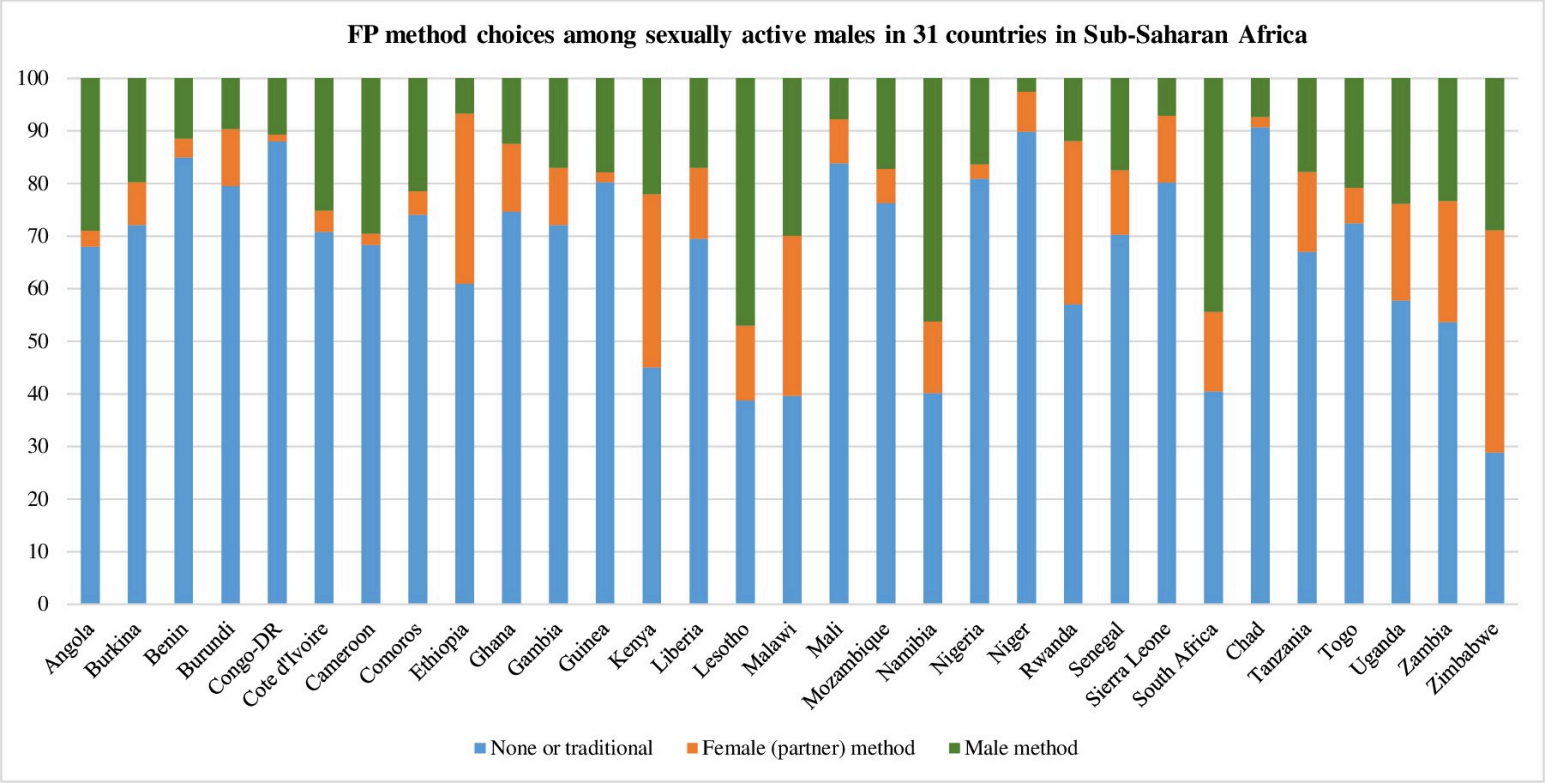
FP method choices among sexually active reproductive age men in Sub-Saharan Africa, by country.

**Fig 4:**
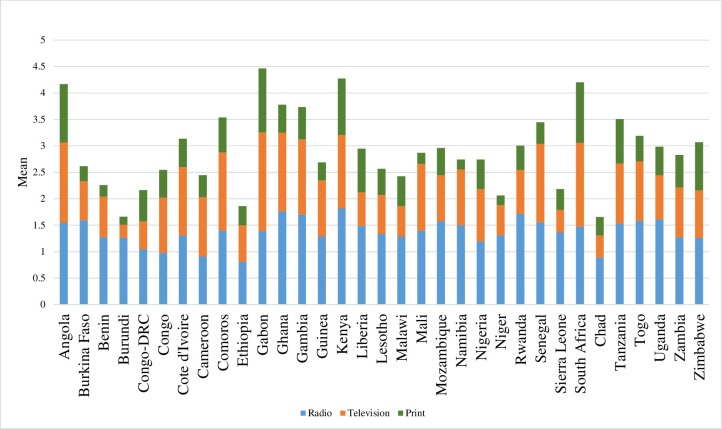
Mean frequency of exposure to radio, television and print media by country.

**Table 2** presents a descriptive summary of pooled sample characteristics across the 31 countries, while **Figs 1–4** present the country-specific distributions of the outcome and primary predictor variables. The average age of the sample was 33.9 years, representing the 1986–1995 birth cohort. Majority (78.3%) of respondents had at least primary school education, with 38% secondary school education attainment. Majority of respondents resided in rural areas (58.1%) and 38.4% resided in the poorest/poor households. Over two thirds (67.3%) of respondents were currently married or cohabiting, and 41.6% had 1–4 children. Overall **(Table 2)**, the FP knowledge was high (97.3%), ranging from 80% in Chad to 99.9% in Zimbabwe (**Fig 1**).

More than two thirds of respondents (73.6%) disagreed with the statement that FP is a woman’s business, but a comparatively lower proportion of men (61.2%) disagreed with the statement that women who use contraception become promiscuous **(Table 2)**.

Angola and Lesotho had the highest proportions of men who endorsed negative attitudes towards FP use, while Rwanda and Ethiopia had the lowest proportions of men who endorsed negative attitudes towards FP use (**Fig 2**).

Almost two thirds of respondents (64.9%) were not using a modern FP method **(Table 2)**. Chad (90.7%) and Niger (89.9%) had the highest proportions of none or traditional method use, while Zimbabwe (23.1%) had the lowest proportion of none or traditional method use (**Fig 3**).

A larger proportion of respondents relied on men’s methods (20.4%) compared to women’s (partner) methods (14.7%). The most common women’s (partner) method choice was injectables, ranging from 1.2% in Congo Democratic Republic to 42.3% in Zimbabwe. The most common men’s method choice was condoms, ranging from 2.6% in Niger to 47% in Lesotho. Over half of respondents (57.3%) listened to radio at least once a week, 39.4% watched television at least once a week, and 20.2% read a newspaper or magazine at least once a week **(Table 2)**.

Gabon had the highest average exposure to all three types of mass media (combined), while Chad had the least mean exposure of all three types of mass media (**Fig 4**). Men in Kenya (1.8) and Rwanda (1.7) reported the highest exposure to radio, while men in Ethiopia (0.8), Cameroon (0.9) and Congo (0.9) reported the lowest exposure to radio. Men in Gabon (1.9) and Burundi (0.3) had the highest and lowest exposure to television, respectively. The highest levels of exposure to print media (i.e., newspapers and magazines) were observed in Gabon (1.2), South Africa (1.1) and Angola (1.1), while the lowest levels of exposure to print media were observed in Burundi (0.1), Benin (0.2), Niger (0.2), Namibia (0.2) and Mali (0.2).

### Relative contribution of mass media exposure to FP knowledge, attitudes and method choice

#### FP knowledge

In the multivariable analyses (**Table 3**), men reporting medium (OR = 2.37; 95% CI: 2.1–2.7) and high (OR = 3.23; 95% CI: 2.9–3.6) frequency of exposure to radio were 2–3 times more likely to know at least one modern FP method compared to men without any exposure to radio. A similar pattern was observed with medium (OR = 2.63; 95% CI: 2.2–3.1) and high exposure to television (OR = 3.18; 95% CI: 2.7–3.8). Medium exposure to print was significantly associated with 49% increase in the odds of knowing at least one modern FP method (OR = 1.49; 95% CI: 1.2–1.9), but the high exposure to print was not significantly associated with FP knowledge. This multivariable model accounted for 12.5% of the variance in FP knowledge (**Table 4**), with the largest relative contribution from radio (26.1%), followed by television (21.4%) and education (20.1%); print contributed 8% to the total variance.

#### FP attitudes

Relative to men reporting low exposure to radio, medium- (OR = 0.81; 95% CI: 0.77–0.85) and high- (OR = 0.75; 95% CI: 0.72–0.78) levels of exposure to radio were significantly associated with lower odds of agreeing with the statement that FP is a woman’s business. Similarly, medium (OR = 0.95; 95% CI: 0.9–0.99) and high (OR = 0.93; 95% CI: 0.89–0.97) levels of exposure to radio were significantly associated with lower odds of agreeing with the statement “women who use FP become promiscuous”. For both attitudinal statements, medium levels of exposure to television (OR = 0.83; 95% CI: 0.78–0.87 and OR = 0.91; 95% CI: 0.87–0.96, respectively) had a greater suppressive effect on FP attitudes compared to high levels of exposure to television (OR = 0.90; 95% CI: 0.86–0.95 and OR = 1.01; 95% CI: 0.96–1.06, respectively). A somewhat similar pattern occurred with respect to frequency of exposure to print. Medium levels of exposure to print were associated with lower odds of agreeing with the statement “FP is a woman’s business” (OR = 0.83; 95% CI: 0.79–0.87), while high levels of exposure were marginally associated with higher odds of endorsing this statement (OR = 1.06; 95% CI: 1.0–1.13). Medium levels of exposure to print (OR = 0.82; 95% CI: 0.79–0.86) had a greater suppressive effect on the attitudinal statement that women who use FP become promiscuous compared to high levels of exposure to print (OR = 0.88; 95% CI: 0.84–0.93).

The multivariable model accounted for 2.77% of the variance in statement that “FP is a woman’s business” (**Table 4**), with the largest relative contribution from education (29.7%), followed by household wealth (23.5%). Lastly, increasing levels of education were significantly associated with lower odds of endorsing both attitudinal statements on FP use. The relative contributions of mass media exposure were comparatively smaller than education, with a largest contribution from frequency of exposure to radio (15.2%) compared to television (7.7%) and print (6.2%).

The multivariable model accounted for 1.67% of the variance in the attitudinal statement that “women who use FP become promiscuous”, with the largest relative contribution from household wealth (23%), followed by birth cohort (21.8%) and education (19.9%). The relative contributions of mass media exposure were comparatively smaller, with a larger contribution from frequency of exposure to print (10.7%) relative to radio (5.2%) and television (2.9%).

#### FP method choice

Only a high frequency of exposure to radio was significantly associated with use of a woman (partner) modern method (OR = 1.10; 95% CI: 1.03–0.17), suggesting a potential threshold effect. Frequency of exposure to radio was not significantly associated with the man’s method choice. Relative to low levels of exposure to television, respondents with medium (OR = 1.21; 95% CI: 1.14–1.29) and high (OR = 1.23; 95% CI: 1.15–1.31) levels of exposure to television were significantly more likely to use a woman (partner) modern method. Similarly, men reporting medium (OR = 1.43; 95% CI: 1.26–1.41) and high (OR = 1.63; 95% CI: 1.54–1.73) levels of exposure to television were also more likely to use a men’s FP method compared to men with low exposure to television. A similar pattern occurred with regard to frequency of exposure to print.

The multivariable logistic model accounted for 6.47% of the total variance in use of women’s methods (**Table 4**), with the largest contributions from country effects (39.1%) and respondents’ marital status (26.6%). Mass media exposure accounted for a comparatively smaller contribution to the variance, with a greater contribution from print (8%) compared to radio (5.6%) and television (0.9%). For men’s FP methods, the multivariable logistic model accounted for 17.55% of the total variance (**Table 4**), with the largest contributions from respondents’ marital status (36.9%), followed by birth cohort (22.9%) and education attainment (12.4%). Mass media exposure accounted for a comparatively smaller contribution to the variance, with a greater contribution from television (8%) compared to print (3.2%) and radio (0.5%). Supplemental analyses with country-specific models largely confirmed the direction of these findings within individual countries.

## Discussion

The paper sought to provide estimates of the relative impact of different types of traditional mass media–radio, television and print–on FP knowledge, attitudes and method choices among reproductive age men in SSA. Findings indicate that mass media exposure positively influences knowledge, FP attitudes and method choice. Mass media exposure was significantly associated with increased FP knowledge and a lesser likelihood of endorsing negative attitudes towards family planning. Mass media exposure also increased the likelihood of modern FP method. However, the effects of each individual medium varied by outcome, suggesting that the effects of mass media on family planning depend on the outcome of interest. Education, household wealth and marital status were also very influential in predicting the different outcomes, more often with a stronger effect than mass media exposure.

These study findings are consistent with the existing literature on the effect of mass media exposure on FP knowledge, attitudes and FP use [23, 26–31]. This paper builds on this literature by highlighting how the different types of mass media differentially influence FP method choice. Specifically, television appears to have a greater impact of the likelihood of using a man-driven method compared to radio and print, which are more impactful on the likelihood of using a woman- (partner) driven method. Mass media exposure influences FP attitudes and behavior by either providing new information or alternative forms of behavior, or by altering ideation pathways that shape consumer’s aspiration and self-identity [21–24]. It is beyond the scope of this paper to evaluate the mechanisms underlying the associations between mass media exposure and FP but these findings provide additional support for the impact of ongoing FP promotion efforts on men’s uptake of FP [36].

These findings highlight the enduring impact of socio-economic and programmatic factors on FP uptake in SSA [8, 10, 11]. Education and household wealth are interrelated, and evidence from numerous studies shows education may increase modern FP uptake through desire for smaller family size [11]. The pooled data utilized in these analyses obscures the potential effect of country specific FP programs–the strength and quality of which varies widely across countries included in the analyses. Nonetheless, these findings suggest that short-term investments in FP demand generation programs that are often delivered via mass media can positively influence FP knowledge, attitudes and behaviors. However, the large and sustainable gains in modern FP uptake among men requires investments in education and improved standards of living–the key antecedent factors to fertility aspirations [37, 38]. In SSA, infant and child mortality remains high [39], many families rely on labor intensive subsistence farming that is supported by family labor, and due to lack of government social security programs, children, especially male children, are viewed as a source of security during old age [40]. The relative contribution of country effects on FP method choice underscore the impact of country-level programming on FP method uptake. Access to family planning, method mix, and quality of family planning counseling are examples of elements within country-specific programs that may affect FP method choice.

These analyses are not without limitations. First, these analyses are cross-sectional, so the documented associations do not imply direct causality. Second, they rely on self-report data that are also vulnerable to social desirability bias. However, the large nationally representative population samples used in these aggregate analyses could mitigate the potential threat of individual-level measurement bias on the aggregate estimates. However, this paper does not intend to adjust or minimize this potential bias, because the large population sample used in these aggregate analyses could mitigate the potential threat of individual-level measurement bias on the aggregate estimates. Additionally, this paper does not address the mechanisms underlying the influence of mass media exposure of FP. There is a need for future studies–qualitative and quantitative to explore mass media mechanisms that can shift attitudes and behaviors for sub-populations such as low-education and low-wealth men. Despite these limitations, these findings provide useful insights on the influence of mass media exposure on FP among men in SSA. These findings highlight the impact of mass media-based FP campaigns on men’s FP knowledge, attitudes and to a lesser extent, method choices. However, these effects are relatively small, in comparison to socio-economic and programmatic factors on FP, indicating that significant gains in FP require concerted government in education, improved standards of living, and country specific FP program contexts.

## Conclusion and policy implications

Mass media–a key component of the FP effort index within a country–is a potent tool that allows FP programs to reach a large proportion of the population within a relatively short time. As documented in many previous studies, mass media exposure can increase FP knowledge and uptake, even among men [23, 25–32], but not all mass media channels have the same impact. Our findings suggest that compared to print media, radio and television exposure are effective in increasing FP knowledge, attitudes and behavior. However, television exposure may have a greater effect on FP knowledge than radio exposure, but when it comes to FP attitudes, radio may be more effective than television. The differential effects of television and radio on FP knowledge and attitudes could be attributed to the nature and limitations of the various mass media channels, which influences several attributes of the mass media communication including nature and quality of content that can delivered through these channels, along these other operators e.g. audience segmentation, audience patterns of consumptions and preferences for cognitive processing and use of contextual cues that influence the salience of the content.

Moving beyond knowledge to behavior (in this case uptake of FP)–the ultimate goal of FP programs–requires inputs beyond providing information. In this case, contextual factors such as access and quality of FP program becomes important, as suggested by our findings, which indicate that country specific contextual factors account for 39% of the variance in use of women’s (partner) driven methods–the most common type of FP methods in SSA. Our findings indicate that education and wealth have significant influences on FP attitudes and behavior, thus providing supporting for a multi-sectoral approach to promoting FP uptake. A multi-sectoral approach seems to mainstream implementation of FP policies, interventions and delivery of services across a range of multi-sectoral domains (such as education, gender, finance and internal affairs) to facilitate a holistic contribution to social and economic transformation. The notion of multi-sectoral approach to FP is not novel: it is implied in FP Costed Implementations Plans (CIP) that have been developed across a range of countries in SSA. However, lack of political will or commitment, lack of resources and coordination, and entrenched siloed thinking hamper implementation of these multi-sectoral programs. Additionally, within the FP programming, many of these multi-sectoral programs are skewed towards gender, education and economic empowerment of young girls and women. However, in the current context where young men have high rates of unemployment and in several countries, enrollment in high education is skewed towards women, multi-sectoral FP programs geared towards women only could lead to reverse gender inequities that undermine progress in FP uptake because the men are falling behind in education and economic empowerment. As such, multi-sectoral approaches to seek to engage both genders to ensure gender equity.
